# Matrix Metalloproteinase-9 -1562C/T Promoter Polymorphism Confers Risk for COPD: A Meta-Analysis

**DOI:** 10.1371/journal.pone.0060523

**Published:** 2013-03-28

**Authors:** Lei Chen, Tao Wang, Lian Liu, Yongchun Shen, Chun Wan, Fuqiang Wen

**Affiliations:** 1 Division of Pulmonary Diseases, State Key Laboratory of Biotherapy of China, West China Hospital, West China School of Medicine, Sichuan University, Chengdu, Sichuan, China; 2 Department of Respiratory Medicine, West China Hospital, West China School of Medicine, Sichuan University, Chengdu, Sichuan, China; University of Montana, United States of America

## Abstract

**Background:**

The role of matrix metalloproteinase (MMP) gene polymorphisms in the development of chronic obstructive pulmonary disease (COPD) has been reported with inconsistent results. This meta-analysis was performed to assess the association of MMP-1 -1607G/GG and MMP-9 -1562C/T promoter polymorphisms with COPD susceptibility.

**Methods:**

Published case-control studies from Pubmed and China National Knowledge Infrastructure (CNKI) databases were retrieved. Data were extracted and pooled odds ratios (OR) with 95% confidence intervals (CI) were calculated.

**Results:**

A total of fourteen case-control studies were included in this meta-analysis. Pooled effect size showed an association of MMP-9 -1562 C/T with the risk of COPD (dominant model: TT+CT vs CC; OR: 1.46; 95% CI: 1.02–2.08; p = 0.04). However, no correlation with COPD was revealed in MMP-1 -1607G/GG polymorphism. When stratified by ethnicity, results indicated MMP-1 -1607G/GG (recessive model: G/G vs G/GG+GG/GG; OR: 1.20; 95% CI: 1.01–1.44; p = 0.04) and MMP-9 -1562 C/T (dominant model; OR: 1.66; 95% CI: 1.01–2.71; p = 0.04) were correlated with COPD susceptibility among Caucasians and Asians respectively. According to source of controls, signifiant association of MMP-9 -1562 C/T (additive model: T vs C; OR:1.71, 95% CI: 1.42–2.07; p<0.00001, and dominant model; OR: 1.92; 95% CI: 1.34–2.76; p = 0.0004) with COPD susceptibility was revealed in the subgroup with smoker-based controls. However, in the aforementioned risk estimates, only the association of MMP-9 -1562 C/T (additive and dominant models) with the risk of COPD in the subgroup with smoker-based controls persisted significantly after Bonferroni correction for multiple testing. Moreover, after excluding the studies without Hardy–Weinberg equilibrium and/or with small sample size, the pooled results were robust and no publication bias was found in this study.

**Conclusion:**

This meta-analysis suggests, when using healthy smokers as controls, MMP-9 -1562 C/T, but not MMP-1 -1607 G/GG polymorphism is associated with the risk of COPD.

## Introduction

Chronic obstructive pulmonary disease (COPD) is a worldwide disease, characterized by not fully reversible and progressive airflow limitation. To date, although the underlying mehanisms of COPD have not been fully understood, a genetic predisposition of COPD has been strongly evidenced [1].

Matrix metalloproteinases (MMPs), members of the metzincin group of proteases, have an important part in COPD, owing to their functions as degradation of components of extracellular matrix and regulators of extracellular signaling networks [2]. The role of MMP gene polymorphisms in COPD has been suggested [3], although no MMP polymorphisms have been reported to be correlated with COPD in recent genome-wide association studies (GWAS) [4]. However, i) most of the recent GWAS were performed in the Northern European populations with limited population size and no diverse ethnicities [5], ii) SNPs that possibly had significance but not to reach the genome-wide significant level might be covered in GWAS [6]. Consequently, the lack of GWAS results at the MMP loci for COPD do not preclude the involvement of polymorphisms in MMP genes with COPD.

In the past decade, the association of MMP-1 -1607 G/GG and MMP-9 -1562 C/T polymorphisms in the promotor region with COPD susceptibility have been reported with diverse results [6]–[19]. Ethnic difference, clinical heterogeneity and small sample size in individual studies may account for the inconsistent results with lower statistical powers, and a meta-analysis has been considered to be a useful means to pool the independent statistical powers and thus achieve a quantitative understanding of the associations. Accordingly, in the present study, a meta-analysis was performed to determine the MMP-1 -1607 G/GG and MMP-9 -1562 C/T polymorphisms and the risk of COPD.

## Methods

### Search strategy

Literature search was conducted using the databases, including Pubmed and China National Knowledge Infrastructure (CNKI) (http://www.cnki.net/). CNKI database was found by Tsinghua University of China in 1996 and includes over 8000 Chinese journals covering natural and social sciences. The languages were limited to English and Chinese. The following search terms were utilized: matrix metalloproteinase or MMP, and gene polymorphism or polymorphism, and chronic obstructive pulmonary disease or COPD. The PRISMA flow diagram (Figure S1) and checklist (Table S1) were available as supporting information.

### Data extraction

Two independent reviewers collected the data according to an inclusion and exclusion critera. For inclusion in the meta-analysis, retrieved articles had to inform number of cases and controls, and number of individuals genotype in cases and controls. Exlusion criteria in the meta-analysis were 1) not case-control genetic study, 2) duplicated report, 3) no useful data reported, 4) other MMP polymorphisms except MMP-1 -1607 G/GG and MMP-9 -1562 C/T. Unpublished data were not considered. Disagreement was resolved by discussion before reaching a consensus. If more than one article was published by the same group using the same cases, the study with higher sample size was selected.

### Statistical analyses

In the present meta-analysis, three genetic models were used as follows: 1) additive (for MMP-1 -1607 G/GG, G vs GG and for MMP-9 -1562 C/T, T vs C); 2) dominant (for MMP-1 -1607 G/GG, G/G+G/GG vs GG/GG and for MMP-9 -1562 C/T, TT+CT vs CC); 3) recessive (for MMP-1 -1607 G/GG, G/G vs G/GG+GG/GG and for MMP-9 -1562 C/T, TT vs CT+CC). Categorical variables were presented as odds ratio (OR) with 95% confidence interval (CI). Pooled ORs with 95% CI were calculated and p<0.05 was accepted with statistical significance. Heterogeneity was checked by the Q test. Meta-analysis was done with the fixed-effects model when there was no heterogeneity (p≥0.1). Otherwise, the random-effects model was used. Subgroup analysis was performed by enthinity and source of controls to assess the effect of possible clinical heterogeneity on the summary ORs. Bonferroni correction was utilized for multiple testing. Because multiple comparisons in the two polymorphisms were performed 15 times respectively, the P value lesser than 0.05/15 (0.0033) was accepted for statistical significance after Bonferroni correction. Pearson's χ2 test was used to determine whether the observed frequencies of genotypes in controls conformed to the Hardy–Weinberg equilibrium (HWE). Studies with controls that depart from HWE (p<0.05) and/or with a small number cases (n≤60) were subjected to a sensitivity analysis in order to check the consistency of the overall effect size. Funnel plots, as well as the Begg's rank correlation test and Egger's linear regression test, was used to inspect the potential publication bias, and p<0.05 was considered significant publication bias. All analyses were conducted using Revman 5.0 (Oxford, UK, The Cochrane Collaboration) and Stata 11.0 (StataCorp LP, College Station, TX, USA).

## Results

### Studies included in the meta-analysis

**Figure 1:**
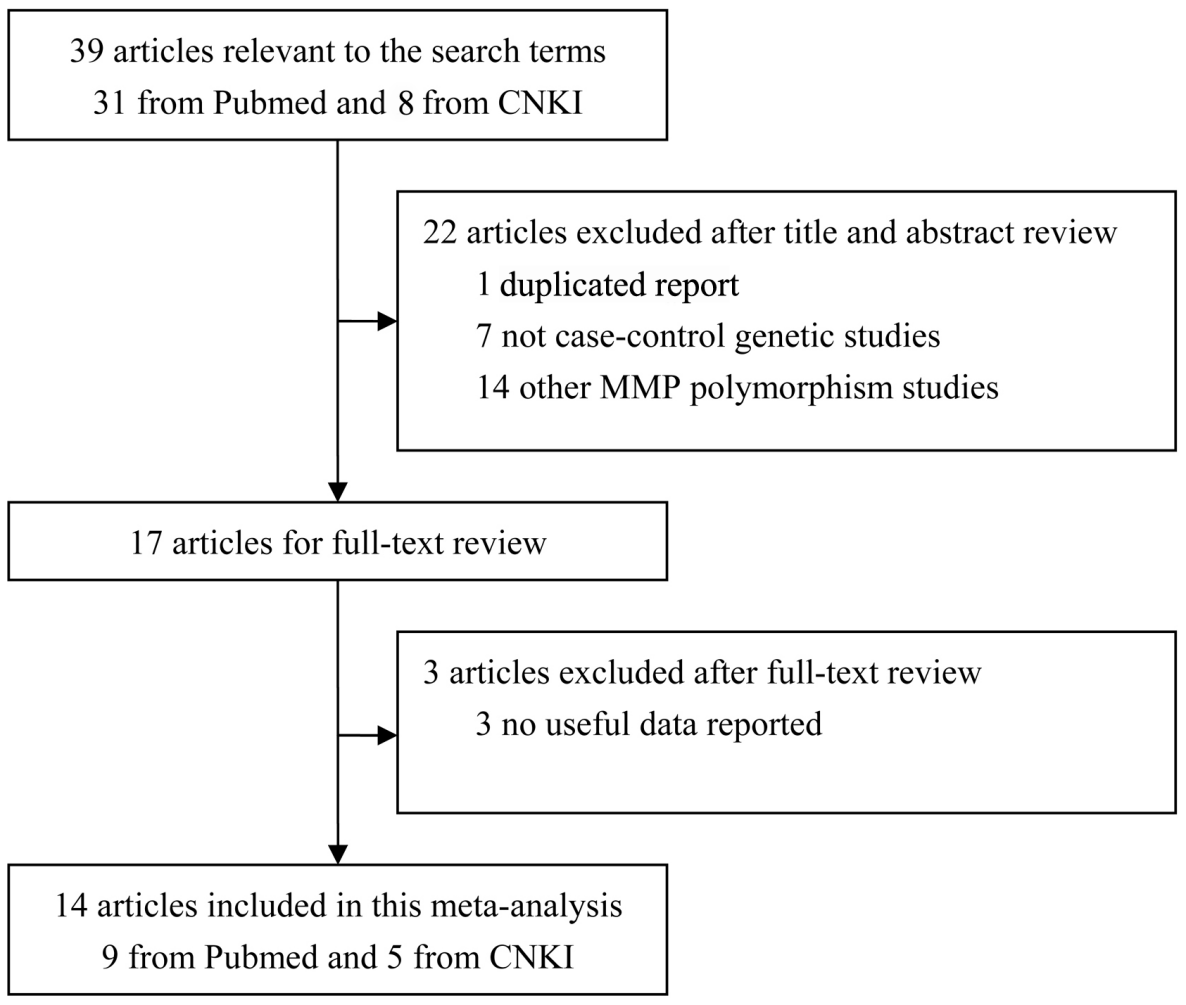
Flow diagram of search process.

Thirty-nine studies were relevant to the search terms. After reviewing the titles, abstracts and articles, twenty-five studies were excluded and thus a total of fourteen studies matched the inclusion criteria (**Figure 1**). Of the fourteen included studies, 1) nine were published in English, and other five in Chinese; 2) three studies examined the MMP-1 -1607G/GG polymorphism, six examined the MMP-9 -1562C/T polymorphism and five examined both the polymorphisms. These studies had been carried out in China, Japan, Korea, USA, Russia, Brazil and Europe. The main features of the studies included in this meta-analysis were presented in **Table 1**.

**Table 1:** Main characteristics of included studies.

Reference	Country	Ethnicity	Genotyping	Source of controls	Case		Control		HWE (*p*)
					N	MAF	N	MAF	
MMP-1 *-1607G/GG*									
Sun et al. 2005	China	Asian	PCR+RFLP	Healthy population	59	0.27	109	0.18	0.002
Zhang et al. 2005	China	Asian	PCR+RFLP	Healthy smokers	147	0.31	120	0.40	0.003
Tesfaigzi et al. 2006	USA	Caucasian	PCR+RFLP	Healthy smokers	109	0.50	218	0.50	0.719
Korytina et al. 2008	Russia	Caucasian	PCR+RFLP	Healthy population	318	0.43	319	0.41	0.858
Cheng et al. 2009	China	Asian	PCR+RFLP	Healthy smokers	184	0.32	212	0.25	0.484
Cai et al. 2010	China	Asian	PCR+RFLP	Healthy smokers	80	0.21	90	0.30	0.008
Lee et al. 2010	Korea	Asian	ABI sequencer	Healthy population	300	0.31	331	0.32	0.126
Haq et al. 2010	Europe	Caucasian	KASPar assay	Healthy smokers	977	0.48	876	0.48	0.447
MMP-9 *-1562 C/T*									
Minematsu et al. 2001	Japan	Asian	PCR+RFLP	Healthy smokers	45	0.24	94	0.16	0.466
Zhou et al. 2004	China	Asian	PCR+RFLP	Healthy smokers	100	0.07	98	0.07	0.995
Zhang et al. 2005	China	Asian	PCR+RFLP	Healthy smokers	147	0.14	120	0.10	0.252
Ito et al. 2005	Japan	Asian	PCR+RFLP	Healthy smokers	84	0.14	85	0.15	0.710
Han et al. 2006	China	Asian	PCR+RFLP	Healthy population	60	0.36	52	0.31	0.782
Tesfaigzi et al. 2006	USA	Caucasian	PCR+RFLP	Healthy smokers	109	0.19	218	0.12	0.865
Korytina et al. 2008	Russia	Caucasian	PCR+RFLP	Healthy population	318	0.12	319	0.13	0.818
Cheng et al. 2009	China	Asian	PCR+RFLP	Healthy smokers	184	0.36	212	0.25	0.484
Schirmer et al. 2009	Brazil	Caucasian	PCR+RFLP	Healthy population	111	0.09	101	0.08	0.676
Lee et al. 2010	Korea	Asian	ABI sequencer	Healthy population	300	0.10	331	0.16	0.869
Hua et al. 2010	China	Asian	PCR+RFLP	Healthy smokers	180	0.10	96	0.08	0.080

HWE: Hardy-Weinberg equilibrium; MAF: minor allele frequency; N: number; PCR: polymerase chain reaction; RFLP: restriction fragment length polymorphism; SNP: single nucleotide polymorphism

### Quantitative synthesis

**Figure 2:**
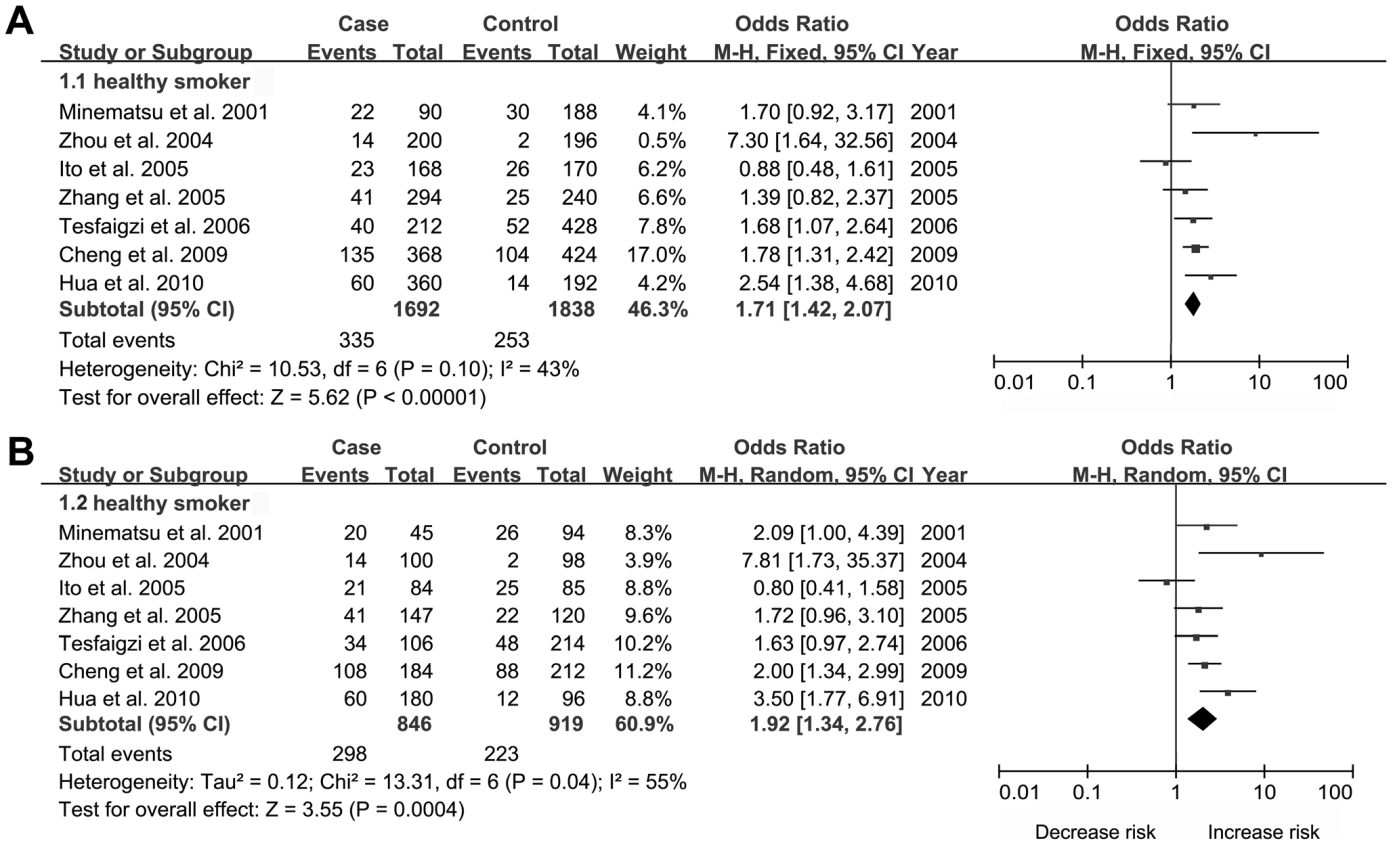
Forest plots of OR with 95% CI for the association of MMP-9 -1562 C/T and COPD risk subanalyzed by source of controls in the additive (A) and dominant (B) genetic models.

Combined results indicated significant association of MMP-9 -1562C/T with an increased risk of COPD using a dominant model (OR: 1.46, 95% CI: 1.02–2.08; p = 0.04), However, no association was found between MMP-1 -1607G/GG and the risk of COPD. In the ethnicity-specific subgroup analysis, MMP-1 -1607G/GG polymorphism had a higher risk for COPD in Caucasians in a recessive model (OR: 1.20, 95% CI: 1.01–1.44; p = 0.04), however, the same pattern of result was found for MMP-9 -1562C/T polymorphism in Asians using a dominant model (OR: 1.66, 95% CI: 1.01–2.71; p = 0.04). Moreover, according to source of controls, signficant association of MMP-9 -1562C/T with COPD risk was revealed in the subgroup with smoker-based controls in the additive (OR:1.71, 95% CI: 1.42–2.07; p<0.00001) (**Figure 2A**) and dominant (OR:1.92, 95% CI:1.34–2.76; p = 0.0004) (**Figure 2B**) genetic models, while there was no association between MMP-1 -1607G/GG and COPD risk. However, after Bonferroni correction, the association of MMP-9 –1562C/T polymorphism in the subgroup of smoker-based controls and COPD risk (additive and dominant models) persisted significantly, while no other associations were found. The main results of pooled estimates in this meta-analysis were presented in **Table 2** **.**

**Table 2:** Main results of pooled estimates in the meta-analysis.

Subgroups	Studies (N)	Effect Size[OR (95%CI); *p-*value; statistic model]		
		Additive model	Dominant model	Recessive model
MMP-1 *-1607G/GG*				
Overall	8	1.00 (0.84–1.20); 0.99; R	1.03 (0.83–1.28); 0.76; R	0.91 (0.66–1.26); 0.58; R
Ethnicity				
Asian	5	0.97 (0.67–1.41); 0.88; R	1.13 (0.76–1.67); 0.54; R	0.68 (0.39–1.20); 0.18; R
Caucasian	3	1.03 (0.93–1.15); 0.57; F	0.92 (0.78–1.08); 0.31; F	1.20 (1.01–1.44); 0.04; F
Source of controls				
Healthy smoker	5	0.93 (0.70–1.22); 0.59; R	0.96 (0.71–1.31); 0.80; R	0.82 (0.50–1.37); 0.46; R
Healthy population	3	1.06 (0.91–1.24); 0.48; F	1.08 (0.87–1.34); 0.48; F	1.06 (0.78–1.43); 0.71; F
MMP-9 *-1562 C/T*				
Overall	11	1.35 (1.00–1.82); 0.05; R	1.46 (1.02–2.08); 0.04; R	1.20 (0.62–2.30); 0.59; R
Ethnicity				
Asian	8	1.44 (0.96–2.17); 0.08; R	1.66 (1.01–2.71); 0.04; R	0.78 (0.31–1.99); 0.61; R
Caucasian	3	1.13 (0.88–1.45); 0.35; F	1.07 (0.81–1.41); 0.66; F	2.23 (0.93–5.33); 0.07; F
Source of controls				
Healthy smoker	7	1.71 (1.42–2.07); <0.0001; F	1.92(1.34–2.76); 0.0004; R	1.55 (0.95–2.53); 0.08; F
Healthy population	4	0.84 (0.69–1.04); 0.11; F	0.82 (0.65–1.04); 0.10; F	0.86 (0.44–1.68); 0.66; F

CI: confidence intervals; F: fixed model; N: number; OR: odds ratio; R: random model; SNP: single nucleotide polymorphism

### Test of Heterogeneity

Significant heterogeneity was revealed between all studies in the meta-analysis, and the source of heterogeneity was detected by ethnicity and source of controls. When stratified by ethnicity, using the three genetic models, no heterogeneity was observed in the studies on both polymorphisms in Caucasians, but not Asians (data not shown). According to source of controls, no significant heterogeneity for MMP-1 -1607G/GG and MMP-9 -1562C/T was revealed in the subgroup with population-based controls, while there was significant heterogeneity in smoker-based control subgroups except for MMP-9 -1562C/T in the additive (I^2^ = 43%, p = 0.10) and recessive (I^2^ = 39%, p = 0.14) models.

### Sensitivity analyses

In the present meta-analysis, five studies (three for MMP-1 -1607G/GG and two for MMP-9 -1562C/T) were lack of HWE and/or smaller sample size, which had a potential to influence the robustness of the present meta-analysis. However, exclusion of these studies did not significantly alter the pattern of the pooled effect size in both polymorphisms (data not shown).

### Publication bias

The funnel plots showed no sigificant asymmetry in studies on both polymorphisms (data not shown). Moreover, publication bias was not suggested by Begg's rank correlation test (MMP-1 -1607G/GG: p = 0.902 for additive model, p = 1.000 for dominant model, p = 0.108 for recessive model; MMP-9 -1562C/T: p = 0.917 for additive model, p = 0.436 for dominant model, p = 0.283 for recessive model) and Egger's linear regression test (MMP-1 -1607G/GG: p = 0.900 for additive model, p = 0.490 for dominant model, p = 0.202 for recessive model; MMP-9 -1562C/T: p = 0.570 for additive model, p = 0.068 for dominant model, p = 0.077 for recessive model).

## Discussion

MMP-1 and MMP-9, major members of MMPs, contribute to the development of cigarette-induced emphysema [20]–[21]. Recently, association of MMP-1 -1607G/GG and MMP-9 -1562C/T with COPD caught more attention, because the two promoter polymorphisms had substantial effects on gene expression and/or function [22]–[24]. However, conflicting results about the association were reported in the past decade.

In this meta-analysis, MMP-9 -1562C/T, but not MMP-1 -1607G/GG, was found to be correlated with COPD in a dominant model, indicating individuals carrying at least a T allele may have a higher risk for COPD than those carrying C homozygote. Interestingly, in the included studies, minor allele frequency (MAF) for MMP-1 -1607 G/GG was dramatically different between Asians and Caucasians, while MAF for MMP-9 -1562C/T in Caucasians was within the range of that in Asians, which manifested MMP-1 -1607 G/GG polymorphism for COPD was probably in an ethnicity-specific pattern and the variability of MMP-9 -1562C/T for COPD might be across different ethnicities or geographic locations. However, significant heterogeneity was revealed between all studies. To identify the source of heterogeneity, subgroup analysis was performed according to ethnicity and source of controls. Pooled ORs by meta-analysis suggested an ethnicity-dependent results that MMP-1 -1607G/GG in Caucasians (recessive model) and MMP-9 -1562C/T in Asians (dominant model) were risk factors for COPD. Noticeably, no heterogeneity was observed in the studies on both polymorphisms in Caucasians. Futhermore, according to source of controls, there was a signficant association of MMP-9 -1562C/T with COPD risk in the subgroup with smoker-based controls, with no heterogeneity in the additive and recessive models. However, the same pattern of result was not observed in the analysis of MMP-1 -1607G/GG polymorphism. These data suggested different ethnicity and source of controls may account for the overall heterogeneity. Noticeably, after Bonferroni correction for multiple testing, only the association of MMP-9 -1562C/T polymorphism (additive and dominant models) in the subgroup of smoker-based controls and COPD risk persisted significantly, while no other associations were found. In the assessment of the robustness of this meta-analysis, no significant alteration of the total effect size in both polymorphisms was detected with exclusion of the studies for departure from the HWE and/or smaller sample size. Publication bias was not suggested in the present study, possibly owing to the deliberate search strategy and data extraction.

However, some limitations should be considered. First, the pooled estimates in this meta-analysis were not based on adjustment by confused factors, such as sex, age, smoking history. Second, some of the studies had small sample size and did not have adequate power to detect the risk for COPD. Third, lack of the original data in the studies limited our further analysis of the potential interactions between gene and gene, or gene and environment, which might modulate COPD risk.

In conclusion, although the pooled estimates should be interpreted with caution, our meta-analysis suggests, when using healthy smokers as controls, MMP-9 -1562C/T, but not MMP-1 -1607G/GG polymorphism is associated with the risk of COPD. However, large sample size studies with unbiased genotyping methods, standardized defined COPD cases and matched controls in different populations, and more detailed data about individual and environment are warranted. Additionally, investigators should pay more attention to the combined effects on gene-gene and gene-environment interactions that may lead to a better understanding of the association of MMP gene polymorphisms with COPD risk.

## Supporting Information

Figure S1 The PRISMA flow diagram.
(DOC)

Table S1 PRISMA Checklist.
(DOC)
