# Supplementary material for: Matrix Metalloproteinase-9 -1562C/T Promoter Polymorphism Confers Risk for COPD: A Meta-Analysis
Source: PLoS One. 2013 Mar 28;8(3):e60523. doi: 10.1371/journal.pone.0060523 (PMC3610819; doi:10.1371/journal.pone.0060523)
Supplement: Figure S1 — The PRISMA flow diagram. (DOC) [file pone.0060523.s001.doc]

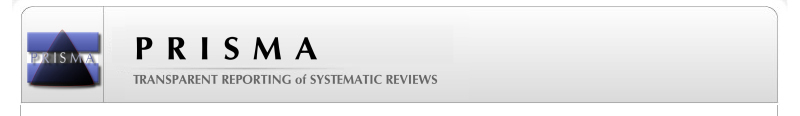
**PRISMA Flow Diagram**

**Screening**

**Included**

**Eligibility**

**Identification**

Records identified through database searching
(n =39)

Additional records identified through other sources
(n =0)

Records after duplicates removed
(n =38)

Records screened
(n = 38 )

Records excluded
(n = 21)

Full-text articles assessed for eligibility
(n = 17)

Full-text articles excluded, with reasons
(n =3)

Studies included in qualitative synthesis
(n =14)

Studies included in quantitative synthesis (meta-analysis)
(n = 14 )
